# Omega-3 Fatty Acids Improve Chronic Kidney Disease—Associated Pruritus and Inflammation

**DOI:** 10.3390/medicina58060796

**Published:** 2022-06-13

**Authors:** Ya-Ling Lin, Chia-Liang Wang, Kai-Li Liu, Cheng-Nan Yeh, Tsay-I Chiang

**Affiliations:** 1 Department of Nursing, Hungkuang University, Taichung 403, Taiwan; poiuppoiup@gmail.com; 2 Department of Nutrition, Chung Shan Medical University, Taichung 402, Taiwan; hollyspirit31@yahoo.com.tw (C.-L.W.); kaililiu@csmu.edu.tw (K.-L.L.); 3 Department of Nephrology, Kuang-Tien General Hospital, Taichung 433, Taiwan; 4 Department of Pediatric Cardiology, Chong-Yo United Clinic, Tainan 712, Taiwan; minami802397@gmail.com

**Keywords:** chronic kidney disease, hemodialysis, pruritus, inflammation, skin moisture

## Abstract

*Background and Objectives*: Chronic kidney disease-associated pruritus (CKD-aP) is a common symptom in hemodialysis patients. A frequent and intense itching sensation largely torments patients, impacts quality of life outcomes, and it has an independent association with mortality. The objective of this study is to investigate the effects of oral supplementation with omega-3 polyunsaturated fatty acid (omega-3 PUFA) on circulating interleukin-6 (IL-6), cardiometabolic parameters, skin moisturization, and the consequent symptoms of pruritus in hemodialysis patients. *Materials and Methods*: Volunteers on maintenance hemodialysis with very severe pruritus symptoms were enrolled in this prospective cohort study. Subjects were instructed to consume 1000 mg fish oil once daily for 3 months. Pruritus scoring, skin moisture, plasma IL-6, and cardiometabolic parameters were measured at baseline, and at the first, second, and third month post-supplementation with fish oil for assessment of the clinical significance. *Results*: A total of 27 patients who had a mean age of 67.33 ± 11.06 years and 3.98 ± 3.23 years on hemodialysis completed the study. Supplementation with omega-3 PUFA significantly decreased IL-6 levels (*p* < 0.001), but increased the levels of c-reactive protein (CRP) (*p* < 0.05). Evaluation of the cardiovascular risk showed significant (all *p* < 0.001) decreases in the total cholesterol (CHO), low-density lipoprotein (LDL), and triglycerides (TG) levels, and an increase in the high-density lipoprotein (HDL) level. A significant decrease in plasma creatinine (CR) was observed (*p* < 0.001), but the decrease was limited. Supplementation with omega-3 PUFA significantly improved (all *p* < 0.001) skin hydration on both the face and arms, as well as disease-related symptoms of pruritus. Conclusion: Omega-3 PUFA supplementation improved inflammation, renal function, cardiovascular parameters, dry skin conditions, and the consequent symptoms of pruritus in hemodialysis patients.

## 1. Introduction

Chronic kidney disease-associated pruritus (CKD-aP) is a common, distressing, and complicated symptom found in patients with advanced CKD or end-stage renal disease (ESRD). This symptom affects 15–49% of pre-dialysis CKD patients and 50–90% of dialysis patients, depending on the type of dialysis [1]. CKD-aP can occur as a generalized itch to a localized itch, most often on the face, back, abdomen, or arms [2]. A prolonged, frequent, and intense itching sensation with daily or near-daily occurrence largely torments patients and impacts quality of life outcomes, such as mental health, sleep quality, and social activity, and has an independent association with mortality [3]. In the past, pruritus was a very common symptom affecting up to 85% of dialysis patients [4]. In recent years, dialysis facilities, therapeutic options, and interventional support have improved and the prevalence of CKD-aP has declined [5]. However, a recent large-scale investigation reported that 40–50% of dialysis patients still suffer from pruritus [6]. Pruritus symptoms remain one of the most troublesome problems in CKD patients.

The etiopathogenesis of CKD-aP has not been fully elucidated. Recent studies suggest it may be caused by multiple or systemic dysfunctions, including immune dysregulation, xerosis, hyperparathyroidism, uremic toxin accumulation, opioid imbalance, and neural dysfunction [5,7]. Immune dysregulation in the background of dysregulated pro-inflammatory factors and inflammatory mediators, such as interleukin-2 (IL-2), prostaglandin E2 (PGE2), serotonin, histamine, proteases, and platelet activating factor, has been generally accepted as the main factor contributing to CKD-aP [2,3,7,8,9,10]. Chronic inflammation is a common comorbidity found in CKD patients [11]. Persistent and low-grade inflammation is also recognized as a main risk factor contributing to the progression of renal disease and both morbidity and mortality in CKD patients [11]. Decreasing inflammation has been reported to ameliorate CKDaP [7,10]. Moreover, abnormal fatty acid profiles and a deficiency of essential fatty acids are generally found in CKD patients [12,13,14]. A low omega-3/omega-6 ratio has been found in peritoneal dialysis patients [13]. Supplementation with essential fatty acids, such as gamma linolenic acid and omega-3 polyunsaturated fatty acids (PUFA), has been reported to ameliorate CKDaP [14,15]. In addition, omega-3 PUFA offers multiple clinical benefits, such as cardiovascular protection, immune modulation, and anti-inflammation, and has few mild, non-distressing adverse effects [14]. Therefore, the hypothesis is that omega-3 PUFA supplementation may be a beneficial option for CKDaP patients. Although some studies have reported that omega-3 PUFA supplementation ameliorates CKDaP, the number of patients was limited and lacked long-term follow-up to validate the therapeutic efficacy of omega-3 PUFA on CKDaP [14].

Omega-3 PUFA supplementation has multiple clinical benefits, as a result it is a prospective option for conventional therapy in CKD patients. The aim of this prospective cohort study was to evaluate the therapeutic efficacy of omega-3 PUFA on CKDaP. We examined the effects of consuming 1000 mg fish oil containing >900 mg eicosapentaenoic acid (EPA) daily in 27 hemodialysis patients for 3 months. We found EPA supplementation significantly ameliorated pruritus. The parameters examined were c-reactive protein (CRP), creatinine (CR), interleukin-6 (IL-6), cholesterol including total cholesterol (CHO), high-density lipoprotein (HDL), low-density lipoprotein (LDL), moisture levels of the face and arms, and the pruritus score.

## 2. Materials and Methods

### 2.1. Subjects

The trial was designed as a prospective cohort study. Volunteers of both sexes, aged between 20–65 years with a high severity of pruritus who had undergone hemodialysis 3 times a week for over 6 months, and had a dialysis adequacy (Kt/V value) of blood urea nitrogen (BUN) greater than 1.2 were recruited from a dialysis facility in Kuang Tien General Hospital, Shalu District, Taichung, Taiwan. Patients with dermatological conditions diagnosed by a dermatologist and allergic reactions to seafood, omega-3 fatty acids, and other related products were excluded. Patients who had medical records for pruritus treatments including topical treatments within 2 weeks, oral treatments within 1 month, and narrowband ultraviolet B phototherapy within 6 months were excluded. Eligible patients who had not used fish oil were identified and approached for consent to partake in the trial. A pruritus questionnaire, measurement of skin moisture, and blood withdrawal were requested for further analyses at baseline, and at the first, second, and third month post-supplementation.

A total of 45 volunteers were recruited, and 31 patients followed the criteria as described above, with two patients dropping out voluntarily and two subjects who passed away during the trial.

### 2.2. Supplementation with Omega-3 Polyunsaturated Fatty Acid

Subjects were instructed to consume one capsule containing 1000 mg fish oil with >900 mg EPA (PURE-EPA90 capsule, Chen Hua Biotech Co., LTD, Taoyuan, Taiwan) once daily for 3 months. Subjects were withdrawn if any symptoms occurred, including allergic or adverse reactions to fish oil. None of the patients showed allergic or adverse reactions during the trial.

### 2.3. Pruritus Questionnaire

The overall pruritus score was determined by a subjective questionnaire with the 5-D itch scale developed by Elman et al. for assessment of CKDaP [16]. The questionnaire covered the criteria of the itching sensation, including duration, degree, direction, distribution, and disability, such as spleen disruption and social dysfunction. Severity of pruritus scoring was defined as non-present (≤8), mild (9–11), moderate (12–17), severe (18–21), and extreme (≥22). Patients answered the same questionnaire at baseline and post-supplementation.

### 2.4. Measurement of Skin Moisture

Moisture levels of the face and arms were measured using Moisturemeter SC (Delfin Technologies Ltd., Kuopio, Finland) to evaluate the skin surface hydration level. The device detects the tissue dielectric constant (TDC) value using a 1.25 MHz electromagnetic probe to determine conductivity in the epidermal layer, while the dry layer of the stratum corneum acts as an insulator. Average TDC values at baseline and post-supplementation were determined by constantly measuring five different sites on each surface of the face and the arms.

### 2.5. Biochemistry and Interleukin-6 Analysis

The collected plasma from patients at baseline and post-supplementation were measured for concentrations of c-reactive protein (CRP), creatine (CR), total cholesterol (CHO), high-density lipoprotein (HDL), low-density lipoprotein (LDL), and interleukin-6 (IL-6). Concentrations of CRP, CR, CHO, HDL, and LDL were detected by routine biochemistry analyses using clinical biochemistry systems (Beckman Coulter, Inc., Brea, CA, USA) at the clinical laboratory in the Kuang Tien General Hospital. The concentration of serum IL-6 was detected by using an IL-6 ELISA kit (Biolegend, San Diego, CA, USA). All of the sera were stored at −80 °C until analysis.

### 2.6. Statistics

Repeated measure ANOVA and a Chi-squared test were used to determine significant differences across each of the time points within baseline and post-supplementation. Analyses were carried out using SPSS analytic software (International Business Machines Corporation, Armonk, NY, USA). A *p* value of <0.05 was considered statistically significant.

## 3. Results

The demographic and baseline characteristics of the participants are presented in Table 1, Table 2 and Table 3. A total of 27 subjects, including 13 males and 14 females, with an average age of 67.33 ± 11.06 years and an average of 3.98 ± 3.23 years of hemodialysis completed the prospective cohort study (Table 1). Following the supplement of fish oil, no significant changes, including BMI, serum total calcium, serum phosphorus, and serum iPTH, were found in comparison with the baseline and the third month (Table 2). These participants had an average pruritus score of 22.85 ± 4.73 at baseline (Table 3). Regarding the severity of pruritus, 11 (40.7%) patients were severe and 16 (59.3%) patients were extreme at baseline (Table 4). The baseline blood biochemical profiles, including CRP, CR, CHO, HDL, LDL, TG, and IL-6, are shown in Table 3. The moisture levels of the face and arms, represented as TDC values, are shown in Table 3. Each parameter described above was examined at baseline, and at the first, second, and third month of post-supplementation with 1000 mg of fish oil daily.

Supplementation with fish oil once daily significantly ameliorated CKDaP in hemodialysis patients (*p* < 0.001), with average decreasing pruritus scores of 19.67 ± 6.14, 15.70 ± 4.53, and 14.11 ± 7.14 at the first, second, and third month, respectively (Table 3). The severity of pruritus levels decreased following supplementation with fish oil over time (Table 4).

To evaluate inflammation following fish oil supplementation, we examined circulating inflammatory indicators, including CRP and IL-6 (Table 3). We found supplementation with fish oil improved the serum CRP levels and decreased the concentration averages (0.40 ± 0.29, 0.35 ± 0.22, 0.31 ± 0.25, and 0.29 ± 0.19 mg/dL) from baseline to the first, second, and third month, respectively (*p* < 0.05, Table 3). The serum IL-6 levels also significantly decreased (averages: 21.81 ± 27.48, 11.04 ± 9.98, 7.73 ± 7.70, and 3.72 ± 3.06 pg/mL) from baseline to the first, second, and third month, respectively (*p* < 0.001, Table 3). Moreover, we found that fish oil supplementation improved renal function, as represented by the decreasing concentration of plasma CR (averages: 10.52 ± 2.50, 10.46 ± 2.14, 10.07 ± 2.32, and 10.28 ± 2.20 mg/dL) from baseline to the first, second, and third month, respectively (*p* < 0.001, Table 3).

To further examine the effects of fish oil supplementation on hematological factors, we detected the blood CHO, HDL, LDL, and TG (Table 3). Supplementation with fish oil significantly decreased the CHO concentration (*p* < 0.001) over time, as represented by the average concentrations of 168.74 ± 33.22, 161.74 ± 29.40, 150.48 ± 39.41, and 169.85 ± 37.02 mg/dL, respectively (Table 3). The average concentrations of HDL increased significantly (33.00 ± 11.11, 34.22 ± 12.11, 35.52 ± 12.40, and 37.04 ± 10.33 mg/dL, respectively) at each time point respectively (*p* < 0.001) (Table 3). The average concentrations of LDL decreased over time (89.26 ± 28.54, 83.96 ± 25.61, 82.44 ± 22.83, and 90.96 ± 28.72 mg/dL, respectively) (*p* < 0.001) (Table 3). The blood TG levels also improved significantly, demonstrated by the decreasing concentrations at each time point (averages: 179.78 ± 109.54, 179.89 ± 99.07, 130.48 ± 59.73, and 142.59 ± 96.28 mg/dL, respectively) (*p* < 0.001) (Table 3).

Dry skin is a common problem in hemodialysis patients. To further examine whether fish oil supplementation would improve the skin condition in hemodialysis patients, we detected skin surface hydration levels represented by TDC values using a non-invasive moisturemeter. Higher TDC values represented higher hydration of the skin surface. We found that fish oil supplementation significantly improved skin dryness (average TDC values: 11.66 ± 4.51, 15.26 ± 4.85, 21.18 ± 6.39, and 26.02 ± 11.23 on arms, and 24.76 ± 12.03, 33.81 ± 13.82, 46.53 ± 14.85, and 47.51 ± 18.87 on face at baseline and the first, second, and third month, respectively) (*p* < 0.001, Table 3).

Evaluation of the pruritus levels showed that the ratio of severity improved significantly from the first month post-supplementation. Higher pruritus levels decreased in this prospective cohort study using post-supplementation with fish oil (Table 4).

## 4. Discussion

The clinical management of pruritus remains a significant issue and is one of the most difficult challenges in CKD patients. The unclear and complicated pathology of CKDaP and its systemic dysregulation makes it very difficult to develop a reliable therapeutic treatment or strategy. The usage of several treatments for CKDaP, such as gabapentin, nalfurafine, thalidomide, cholestyramine, and ondansetron, are limited because of their adverse effects [3,17]. Moreover, dupilumab, an antibody-based drug that acts as an antagonist of IL-4Rα, modulating the signaling of both the IL-4 and IL-13 pathways to ameliorate allergic reactions, has been suggested to treat CKDaP [18]. However, the cost of dupilumab is high and its potential adverse effects make it unfavorable for the treatment of CKDaP [18,19]. Therefore, further investigation of therapeutic approaches with fewer side effects and affordable prices are needed for CKDaP patients. The consequent effect of omega-3 PUFA in ameliorating CKDaP might occur through the improvement of the inflammatory markers, including decreased levels of IL-6 and CRP, and enhancement of the skin moisture. Furthermore, omega-3 PUFA also improved renal function and cardiovascular parameters. In the present study, we suggest that daily supplement of omega-3 PUFA can be beneficial for ameliorating pruritus symptoms, without significant adverse effects, in hemodialysis patients.

Although several hypotheses have been suggested for the pathogenesises of CKDaP, chronic inflammation has been recently recognized as one of the major pathogenic factors [20,21,22,23,24,25]. Kimmel et al. reported that higher levels of serum CRP, IL-6, and TNF-α were found in hemodialysis patients with CKDaP compared with those without CKDaP [21]. Fallahzadeh et al. also reported a significantly higher serum IL-2 level in hemodialysis patients with CKDaP than those without [24]. These two groups also concluded that increased activation of the Th1 cell, a well-known lymphocyte that secretes pro-inflammatory cytokines, might be the pathogenesis of CKDaP [21,24]. Chen et al. reported that a higher severity of pruritus had a significantly higher CRP, and the correlation between CRP level and the severity of pruritus was suggested as an independent predictor of mortality in hemodialysis patients [22]. Schricker et al. revealed that higher inflammatory parameters, including serum CRP and IL-6 levels, had a significant positive association with the severity of CKDaP [23]. A higher serum IL-31 level has also been reported to be associated with pruritus intensity in hemodialysis patients [25]. Reducing inflammatory factors with treatments using anti-inflammatory agents has been reported to ameliorate CKDaP [26,27,28]. Moreover, supplementation with essential oils has been reported to ameliorate CKDaP by decreasing inflammation [14,29,30,31]. In the present study, we found that supplementation with omega-3 PUFA significantly decreased both the plasma CRP and IL-6 levels, as well as the consequent severity of pruritus in hemodialysis patients. The reduced levels of plasma CRP and IL-6 might support the hypothesis of the therapeutic potential of anti-inflammatory agents in CKDaP (Table 3). In addition, we also found that supplementation with omega-3 PUFA mildly improved renal function, as well as decreased the plasma CR concentration (Table 3). Even though we found statistical significance in decreasing the CR levels, the improvement was limited (Table 3).

Chronic inflammation has been recognized as an essential component of CKD and plays an important role in the pathophysiology of disease progression and comorbidity, which results in cardiovascular disease, dysregulated metabolism, and all-cause mortality in CKD patients [11]. Long-lasting systemic inflammation caused by atherosclerosis remains the major risk factor contributing to morbidity and mortality in CKD patients [32]. Although omega-3 PUFA has been well-known for its role in the improvement of cardiovascular disease, its protective effect on CKD patients remains controversial [32]. However, several studies have shown that supplementation with omega-3 PUFA improves cardiometabolic parameters in CKD patients [33]. In this study, we also examined cardiometabolic parameters post-supplementation with omega-3 PUFA. We found that omega-3 PUFA supplementation decreased the plasma levels of CHO, LDL, and TG, and increased the HDL levels (Table 3). Our study suggests that omega-3 PUFA supplementation reduces cardiometabolic risk in hemodialysis patients.

Furthermore, dry skin is an extremely common symptom found in ESRD (end-stage renal disease) patients and has been suggested to be a contributor to CKDaP [7]. Oral supplementation with omega-3 PUFA has been reported to improve dry skin conditions [34]. In the present study, we found that omega-3 PUFA supplementation greatly improved dry skin conditions with 123% and 92% increased hydration on the surface of the arms and face, respectively (Table 3). Our results suggest a potential promising therapeutic of omega-3 supplementation for improving skin conditions and consequent pruritus symptoms.

## 5. Conclusions

In summary, we conducted a prospective cohort study to evaluate the therapeutic potential of omega-3 PUFA supplementation for CKDaP. Our results showed that supplementation with omega-3 PUFA improved inflammation, renal function, cardiovascular parameters, and dry skin conditions, as well as decreasing the number of severe pruritus symptoms in hemodialysis patients (Table 4). In addition, our results showed that omega-3 PUFA can significantly ameliorate the severity of CKDaP after less than 1 month of supplementation (Table 4). However, the number of patients in our cohort was limited. A large-scale study is warranted to prove the hypothesis for the therapeutic efficacy of omega-3 supplementation in CKDaP.

## Figures and Tables

**Table 1:** Baseline characteristics of hemodialysis patients (mean ± SD).

Characteristic	Variable
Age	67.33 ± 11.06
Sex	
Male	13
Female	14
Years on hemodialysis	3.98 ± 3.23

**Table 2:** Comparisons of body mass index, serum ions, and serum intact parathyroid hormone at baseline and at the third month after daily EPA supplementation in hemodialysis patients (mean ± SD).

Characteristic	Baseline	3rd Month	*p* Value
Body height (m)	1.66 ± 0.07	1.66 ± 0.07	^ns^
Body weight (kg)	63.62 ± 10.33	63.64 ± 10.34	0.3643 ^ns^
Body Mass Index (kg/m^2^)	23.09 ± 2.79	23.09 ± 2.79	0.4195 ^ns^
Serum total calcium (mg/dL)	9.12 ± 0.77	9.32 ± 0.60	0.2041 ^ns^
Serum phosphorus (mg/dL)	4.98 ± 1.27	5 ± 1.16	0.9390 ^ns^
iPTH (pg/mL)	184.81 ± 189.87	223.00 ± 275.57	0.3528 ^ns^

iPTH, intact parathyroid hormone. ^ns^, not significant, paired *t*-test.

**Table 3:** Comparisons of Pruritus scores, biochemical profiles, and moisture levels before and after daily EPA supplementation in hemodialysis patients (mean ± SD).

Characteristic	Baseline	1st Month	2nd Month	3rd Month	*p* Value
Pruritus score	22.85 ± 4.73	19.67 ± 6.14	15.70 ± 4.53	14.11 ± 7.14	<0.001 ***
Biochemical profile					
CRP (mg/dL)	0.40 ± 0.29	0.35 ± 0.22	0.31 ± 0.25	0.29 ± 0.19	<0.05 *
CR (mg/dL)	10.52 ± 2.50	10.46 ± 2.14	10.07 ± 2.32	10.28 ± 2.20	<0.001 ***
CHO (mg/dL)	168.74 ± 33.22	161.74 ± 29.40	150.48 ± 39.41	169.85 ± 37.02	<0.001 ***
HDL (mg/dL)	33.00 ± 11.11	34.22 ± 12.11	35.52 ± 12.40	37.04 ± 10.33	<0.001 ***
LDL (mg/dL)	89.26 ± 28.54	83.96 ± 25.61	82.44 ± 22.83	90.96 ± 28.72	<0.001 ***
TG (mg/dL)	179.78 ± 109.54	179.89 ± 99.07	130.48 ± 59.73	142.59 ± 96.28	<0.001 ***
IL-6 (pg/mL)	21.81 ± 27.48	11.04 ± 9.98	7.73 ± 7.70	3.72 ± 3.06	<0.001 ***
Moisture level (TDC value)					
Arms	11.66 ± 4.51	15.26 ± 4.85	21.18 ± 6.39	26.02 ± 11.23	<0.001 ***
Face	24.76 ± 12.03	33.81 ± 13.82	46.53 ± 14.85	47.51 ± 18.87	<0.001 ***

CRP, c-reactive protein; CR, creatinine; CHO, total cholesterol; HDL, high-density lipoprotein; LDL, low-density lipoprotein; TG, triglycerides; IL-6, interleukin-6. * *p* < 0.05 *** *p* < 0.001 was considered statistically significant, repeated measure ANOVA.

**Table 4:** Comparison of pruritus levels before and after daily EPA supplementation in hemodialysis patients (mean ± SD).

Pruritus Level	Non Present	Mild	Moderate	Severe	Extreme	*p* Value
Baseline (%)	0	0	0	11 (40.7)	16 (59.3)	
1st month (%)	0	1 (3.7)	10 (7.0)	9 (33.3)	7 (25.9)	<0.001 ***
2nd month (%)	0	7 (25.9)	11 (40.7)	6 (22.2)	3 (11.1)	=0.042 *
3rd month (%)	7 (25.9)	8 (29.6)	6 (22.2)	1 (3.70	5 (18.5)	=0.015 *

* Significant level *p* < 0.05, *** *p* < 0.001, Chi-squared test.

## Data Availability

The data presented in this study are available in the article.
